# Targeting T Cell Activation and Lupus Autoimmune Phenotypes by Inhibiting Glucose Transporters

**DOI:** 10.3389/fimmu.2019.00833

**Published:** 2019-04-17

**Authors:** Wei Li, Ganlin Qu, Seung-Chul Choi, Caleb Cornaby, Anton Titov, Natalie Kanda, Xiangyu Teng, Haiting Wang, Laurence Morel

**Affiliations:** ^1^Department of Pathology, Immunology and Laboratory Medicine, University of Florida, Gainesville, FL, United States; ^2^Department of Rheumatology, RenJi Hospital South, School of Medicine, Shanghai Jiaotong University, Shanghai, China

**Keywords:** systemic lupus erythematosus, metabolism, glucose transporter, glycolysis, T cells

## Abstract

CD4^+^ T cells have numerous features of over-activated cellular metabolism in lupus patients and mouse models of the disease. This includes a higher glycolysis than in healthy controls. Glucose transporters play an essential role in glucose metabolism by controlling glucose import into the cell from the extracellular environment. We have previously shown that treatment of lupus-prone mice with 2-deoxy-D-glucose, which inhibits the first step of glycolysis was sufficient to prevent autoimmune activation. However, direct targeting of glucose transporters has never been tested in a mouse model of lupus. Here, we show that CG-5, a novel glucose transporter inhibitor, ameliorated autoimmune phenotypes in a spontaneous lupus-prone mouse model, B6.NZM2410.*Sle1.Sle2.Sle3* (Triple-congenic, TC), and in a chronic graft- vs. host-disease (cGVHD) model of induced lupus. *In vitro*, CG-5 blocked glycolysis in CD4^+^ T cells, and limited the expansion of CD4^+^ T cells induced by alloreactive stimulation. CG-5 also modulated CD4^+^ T cell polarization by inhibiting Th1 and Th17 differentiation and promoting regulatory T (Treg) induction. Moreover, CG-5 treatment reduced lupus phenotypes including the expansion of germinal center B (GC B) cells, as well as the production of autoantibodies in both TC mice and cGVHD models. Finally, CG-5 blocked glycolysis in human T cells. Overall, our data suggest that blocking glucose uptake with a small molecule inhibitor ameliorates autoimmune activation, at least partially due to its inhibition of glycolysis in CD4^+^ T cells.

## Introduction

Systemic lupus erythematosus (SLE) is a systemic autoimmune disease, in which autoreactive CD4^+^ T cells play an essential role by providing help to autoantibody-producing B cells both in mice and patients (1). In lupus-prone mice and SLE patients, CD4^+^ T cells present an enhanced cellular metabolism (2–4). Naïve T cells (Tn) or resting T cells have a low energy demand and use mitochondrial oxidative phosphorylation (OXPHOS) to generate ATP for immune surveillance (5), while effector T cells (Teff) or activated T cells show an increase in glycolysis and mitochondrial metabolism to meet the biosynthetic demands (6). Glycolytic utilization in the presence of oxygen was first described in cancer cells as “Warburg Effect” and further found to be essential in activated T cells (7). Glucose uptake provides a key metabolic checkpoint through the Glut family of glucose transporters. Stimulation of CD4^+^ T cells activates the PI3K-AKT pathway, which increases *Glut1* expression, glucose uptake, and metabolism (8). Accordingly, *in vitro*-stimulated murine and human T cells showed an increased expression of *Glut1, Glut3*, and *Glut6* (9, 10). Importantly, transgenic *Glut1* overexpression selectively increased the frequency of Teff cells (9, 11) and follicular helper T cells (Tfh) (12). Conversely, *Glut1* deficiency in CD4^+^ T cells decreased Teff expansion and the ability to induce inflammatory disease *in vivo* (10). Collectively, these results show that glucose uptake, specifically through Glut1, plays an inflammatory role in activated T cells.

The therapeutic potential of targeting immune metabolism has been explored in lupus and as well as in autoimmune arthritis using mouse models (3, 13–16). Treatment with a combination of metformin and 2DG, two metabolic inhibitors that target mitochondrial and glucose metabolism, respectively, reversed lupus phenotypes in lupus-prone mice (3, 14), while treatment with either metformin or 2DG alone could prevent the development of the disease (14). Moreover, 2DG alone reversed the expansion of Tfh cells in multiple models of lupus-prone mice (16). In K/BxN mouse, a mouse model of rheumatoid arthritis, 2DG decreased CD4^+^ T cell and B cell metabolism, and reduced activation of both adaptive and innate immune cells (15). Treatments with low doses of 2DG do not have toxicity effects even with chronic administration (17), but heart vacuolization has been reported in rats treated with a high dose of 2DG (18). Furthermore, 2DG inhibits N-glycosylation (19), which represents a major immunoregulatory mechanism of Teff cell function (20). Although 2DG decreases glucose utilization both by glycolysis and oxidation *in vitro* and *in vivo* (3, 14), it is possible that other functions of 2DG also play a role in reducing autoimmune pathology.

Here, we used a glucose transporter inhibitor, CG-5 that was initially selected as a thiazolidinedione peroxisome proliferator-activated receptor γ agonist (21). After validating that CG-5 inhibits glucose uptake by CD4^+^ T cells, we examined its effect on CD4^+^ T cell activation and polarization *in vitro* as well as in lupus models. CG-5 inhibited glycolysis in activated T cells while promoting fatty acid oxidation and the pentose phosphate pathway. CG-5 inhibited Th1 and Th17 polarization and enhanced Treg differentiation. CG-5 also limited the expansion of CD4^+^ T cells induced by alloreactive stimulation. CG-5 administration ameliorated lupus phenotypes in both spontaneous and induced models of lupus. Finally, CG-5 also inhibited glycolysis in human CD4^+^ T cells. Thus, the effect of this glucose transporter inhibitor is comparable to that of glycolysis inhibitors and underscore the translational potential of inhibiting glucose uptake to treat lupus.

## Materials and Methods

### Mice

TC mice have been described previously (22). C57BL/6J (B6), B6(C)-*H2-Ab1*^*bm*12^/KhEgJ (Bm12), and B6.129P2-*Tcrb*^*tm*1*Mom*^/J (TCRβ KO) mice were originally bought from the Jackson Laboratory. Only female mice were used in this study. Chronic graft- vs. host-disease (cGVHD) was induced in 8–10 weeks-old B6 mice by intravenous injection of 5 × 10^7^ splenocytes from Bm12 mice. For *in vivo* treatment, mice were randomly divided into two groups and gavaged with CG-5 (100 mg/kg per mouse per day) or vehicle alone (0.1% Tween 80 and 15% dimethyl sulfoxide in water). CG-5 was obtained from Ohio State University. All experiments were conducted according to protocols approved by the University of Florida Institutional Animal Care and Use Committee.

### Mouse T Cell Isolation and *in vitro* Activation and Polarization

CD4^+^ T cells were isolated from B6 mice by negative selection with the CD4^+^ T cell isolation kit on the Miltenyi AutoMACS Pro (Miltenyi Biotec). The final purity was >95% CD4^+^ cells. Cells were stimulated in wells pre-coated with 2 μg/ml anti-CD3 (145-2C11, BD Biosciences) with soluble anti-CD28 (37.51, BD Biosciences) at 1 μg/ml for 24 h. For the mixed lymphocyte reaction, CD4^+^ T cells from Bm12 mice were mixed with splenocytes from TCRβ KO mice at a 1:1 ratio in complete RPMI 1640 media for 4 days. Concentrations of drugs were as follows: CG-5 at 2 or 4 μM in 0.1% DMSO; and 2DG at 0.2 mM. For polarization, the Th0 condition corresponds to anti-CD3/anti-CD28 stimulation in complete RPMI 1640. In addition, the Th1-polarizing media contained 10 ng/ml IL-12 (210-12, Peprotech) and 10 μg/ml anti-IL-4 (11B11, BioXcell), the Treg-polarizing media contained 3 ng/ml TGF-ß (100-21, PeproTech), 50 ng/ml IL-2 (402-ML, R&D Systems), 10 μg/ml anti-IFN-γ (XMG1.2, BioXcell), and 10 μg/ml anti-IL-4 antibodies, and the Th17-polarizing media contained 3 ng/ml TGF-ß, 50 ng/ml IL-6 (575704, Biolegend), 300 nM 6-formylindolo (3,2-b) carbazole (Enzo Life Sciences), 10 ng/ml IL-23 (589002, Biolegend), anti-IL-4 and anti-IFN-γ antibodies (10 mg/ml each). Cells were harvested after 5 days and stained for flow cytometry.

### Human CD4^+^ T Cell Cultures

Leukocyte fractions of peripheral blood from healthy donors were obtained through the LifeSouth blood bank (UF IRB approval IRB201700257). PBMCs were isolated with gradient centrifugation using Ficoll-Paque (GE Healthcare). CD4^+^ T cells were isolated with EasySep magnetic isolation (StemCell) with >90% purity. Purified CD4^+^ T cells were cultured for 24 h with plate-bound 1 μg/mL of anti-CD3 (UCHT1, BD Biosciences) and 1 μg/mL anti-CD28 (L293, BD biosciences) Abs in complete RPMI 1640 media in the presence of vehicle, 2 or 4 μM CG5, or 0.2 mM 2DG as described for mouse CD4^+^ T cells.

### Metabolic Measurements

Purified CD4^+^ T cells were analyzed after stimulating with anti-CD3 and anti-CD28 mAbs in the presence of vehicle or CG-5 for 24 h. Oxygen consumption rate (OCR) and extra cellular acidification rate (ECAR) were measured using the XF96 Extracellular Flux Analyzer (Seahorse) under mitochondrial stress test conditions with non-buffered RPMI 1640 medium supplemented with 2.5 μM dextrose, 2 nM glutamine, and 1 uM sodium pyruvate. One micromolar oligomycin, 1.25 μM FCCP, 1 μM rotenone, and 1 μM antimycin A were set as the concentration of the mitochondrial stress assay. The NADP^+^/NADPH ratio was quantified with the NADP/NADPH-Glo™ Kit (Promega) according to the manufacturer's instructions. All experiments were repeated at least one time.

### Flow Cytometry

Single-cell suspensions were prepared using standard procedures from spleen. After RBC lysis, cells were stained in FACS staining buffer (2.5% FBS, 0.05% sodium azide in PBS). Fluorochrome-conjugated Abs, Bcl-6 (K112-91), CD138 (281-2), CD25 (PC61), CD25 (PC61.5), CD279 (RMP1-30), CD4 (RM4-5), CD4 (GK1.5), CD44 (IM7), CD62L (MEL-14), CD69 (H1.2F3), CD95 (15A7), PD-1 (RPM1-30), Foxp3 (FJK-16S), Foxp3 (GL-7), GL7 (GL-7), IFN-y (XMG1.2), IL-17a (TC11-18h10.1), and Ki-67 (SOLA15) were purchased from BD Biosciences, eBioscience, and BioLegend. Intracellular staining was performed with a Fixation/Permeabilization (eBioscience) kit. For cytokine detection, spleen cells were seeded in 96-well plates with 200 μl complete RPMI 1640 media containing Leukocyte Activation Cocktail with GolgiPlug (BD Biosciences) for 4 h. Glucose uptake was measured using 20 nM 2-NBDG (Sigma) at 37°C for 30 min, and total cellular lipid was measured using 2 μM Bodipy (Sigma) for 15 min at 37°C. All samples were acquired on a LSR Fortessa flow cytometer (BD Biosciences) and analyzed with the FlowJo software (Tree Star). The gating strategy is shown in Supplemental Figure 1.

### Gene Expression

RNA was isolated from purified CD4^+^ T cells with the RNeasy Mini Kit (Qiagen) and further used for qRT-PCR using ImProm II Reverse Transcriptase (Promega). SYBR Green Dye (Bio-RAD) was used for quantification of gene expression on the BioRad CFX connect system with primer sequences shown in Supplemental Table 1. The PCR protocol was 30 s at 95°C, 30 s at 60°C, and 30 s at 72°C repeated for 42 cycles. Expression was calculated using the 2^Δ*Cq*^ method with difference in Cq values normalized to the housekeeping gene *Ppia* against the gene of interest.

### ELISA

Anti-dsDNA IgG was measured as previously described (23) in sera diluted 1:100. Relative units were standardized using serial dilutions of a pool of sera from TC mice setting the 1:100 dilution reactivity to 100 U.

### Statistical Analysis

Differences between groups were evaluated by two-tailed statistics: unpaired or paired *t*-tests or one-way ANOVA tests when more than two groups were compared. For *in vitro* experiments, data are presented from one of two independent experiments, each of them with the indicated sample size (*n* = 3–6). The results are expressed as means ± S.E.M. The statistical analyses were performed with the Graphpad Prism 7.0 software. The level of statistical significance was set at ^*^p < 0.05, ^**^p < 0.01, ^***^p < 0.001.

## Results

### CG-5 Blocks Glycolysis in Murine CD4^+^ T Cells

**Figure 1:**
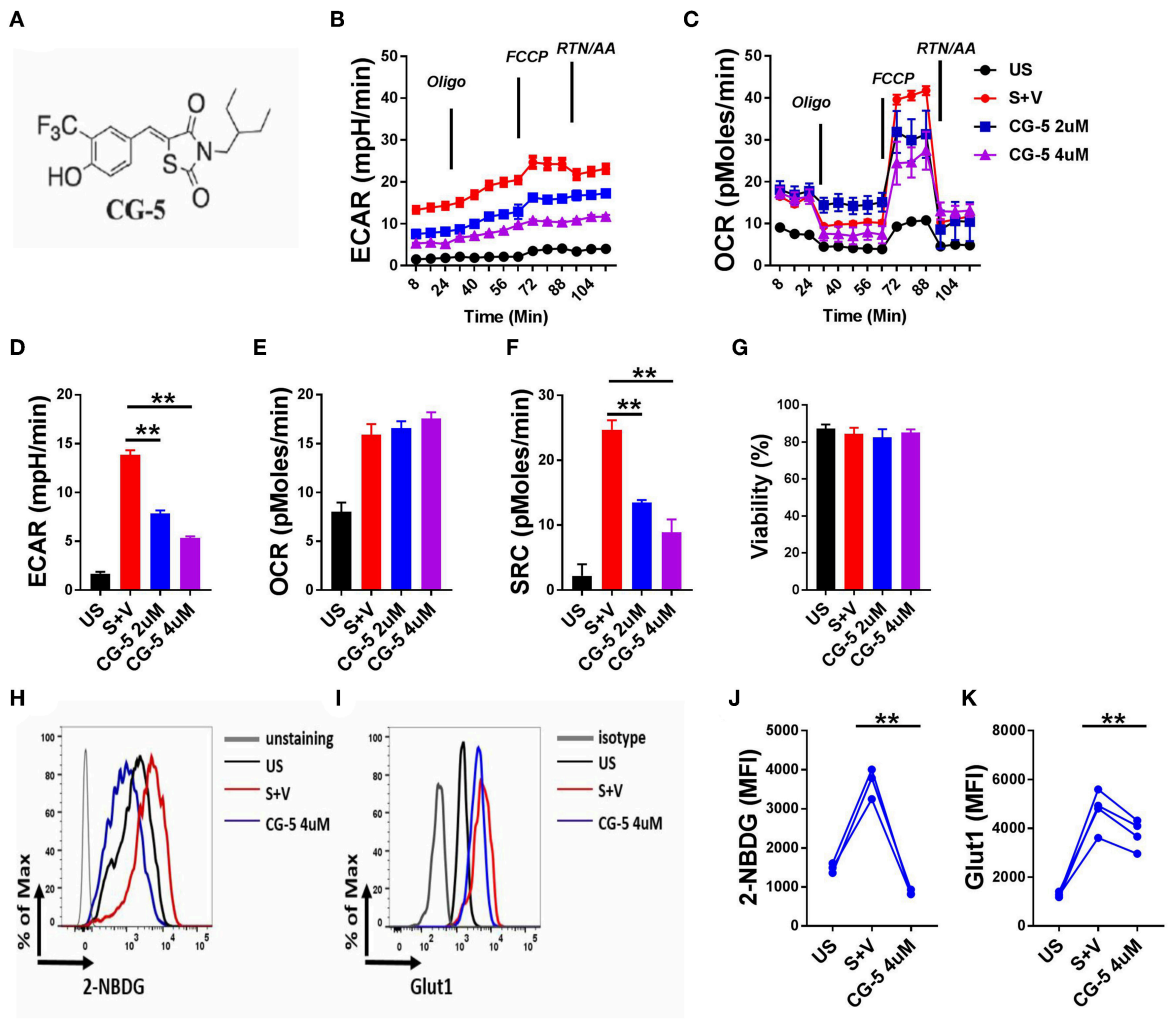
CG-5 inhibits glycolysis in murine CD4^+^ T cells. **(A)** Chemical structure of CG-5. Cells were either unstimulated (US) or stimulated with anti-CD3/CD28 mAbs for 24 h (S) in the presence of vehicle (V), or 2 or 4 μM CG-5. ECAR **(B)** and OCR **(C)** in CD4^+^ T cells under basal conditions and in response to sequential addition of oligomycin, fluorocarbonyl cyanide phenylhydrazone (FCCP), rotenone (Rtn), and antimycin A (AA). Basal ECAR **(D)**, OCR **(E)**, SRC **(F)**, and cell viability **(G)** corresponding to **(B,C)**. 2-NBDG uptake **(H**,**J)** and Glut1 expression **(I**,**K)**. Representative FACS plots **(H**,**I)** of mean fluorescence intensity **(**MFI) quantified in (**J**,**K)**. Mean ± S.E.M of *n* = 3–4 per group compared with S + V values using one-way ANOVA. **p < 0.01.

To address whether CG-5 (Figure 1A) blocks glycolysis in CD4^+^ T cells, we stimulated B6 CD4^+^ T cells with anti-CD3/CD28 mAbs for 24 h and analyzed their OCR, a measure of mitochondrial respiration, and ECAR, a measure of glycolysis. As expected, activated CD4^+^ T cells showed increased ECAR and OCR as compared to unstimulated cells (Figures 1B,C). CG-5 reduced ECAR in a dose-dependent manner (Figure 1D), but did not affect basal OCR (Figure 1E). Spare respiratory capacity (SRC), which is a measure of the ability of the cell to respond to an increased energy demand was also decreased by CG-5 (Figure 1F). Viability of these cells was not affected by CG-5 and was similar in all groups (Figure 1G). Next, we measured glucose uptake using 2-NBDG, a fluorescent glucose analog, in CD4^+^ T cells in the same conditions. As expected, glucose uptake (Figures 1H,J) and Glut1 protein expression (Figures 1I,K) were both upregulated by activation. CG-5 blocked glucose uptake as previously reported in other cell types, and it decreases Glut1 expression at the protein level. However, CG-5 and 2DG treatments showed a variable effect on the gene expression of *Glut1*, and to a lesser extent, *Glut3* or *Glut6*, which tended to be higher than in controls (Supplemental Figure 2). The discrepancy between mRNA and protein expression indicates a potential post-transcriptional regulation of the expression of glucose transporter in response to glycolytic inhibition. Consistent with a decreased glucose flow, the expression of genes involved in glycolysis such as *Hk2, Gapdh, Ldha*, and *Ldhb*, were decreased after treatment with CG-5 in a similar manner as with 2DG (Supplemental Figure 2), most likely as a result of decreased glycolytic activity. Overall, our results demonstrate that CG-5 blocks glucose uptake and glycolysis in activated CD4^+^ T cells.

### CG-5 Promotes Fatty Acid Oxidation and the Pentose Phosphate Pathway in CD4^+^ T Cells

**Figure 2:**
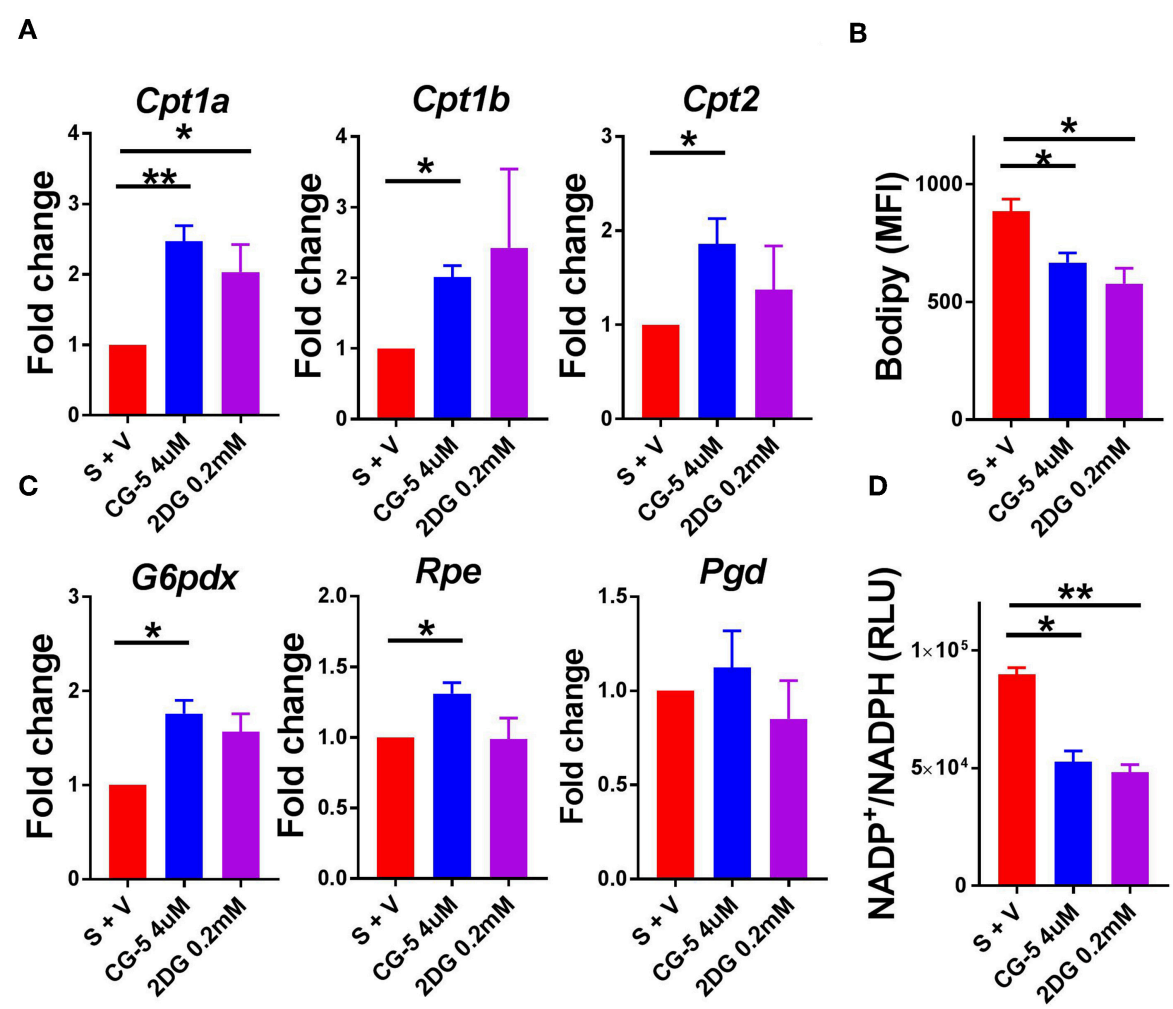
CG-5 promotes FAO and PPP activation in CD4^+^ T cells. B6 CD4^+^ T cells were stimulated with anti-CD3/CD28 mAb in the presence of vehicle (S + V), CG-5, or 2DG for 24 h. **(A)** Relative expression of FAO genes, *Cpt1a, Cpt1b*, and *Cpt2*, normalized to vehicle-treated group. **(B)** Cellular lipid measured as Bodipy staining by flow cytometry. **(C)** Expression of PPP genes, *G6pdx, Rpe*, and *Pgd*, normalized to vehicle-treated group. **(D)** NADP^+^/NADPH ratio in cell lysates (RLU, Relative light unit). Mean ± S.E.M of *n* = 3–6 per group. *p < 0.05; **p < 0.01.

Since glucose uptake was blocked by CG-5 in activated CD4^+^ T cells, we next studied which energy source fueled cellular metabolism when glycolysis was inhibited. The expression of genes involved in the fatty acid oxidation (FAO) pathway, *Cpt1a, Cpt1b*, and *Cpt2*, was increased after CG-5 treatment, indicating a skewing to FAO when glycolysis was inhibited (Figure 2A). CG-5 also reduced the amount of cellular lipid droplets stained by Bodipy (Figure 2B), suggesting an increased utilization of fatty acids. The pentose phosphate pathway (PPP) is a shunt of glycolysis to generate NADPH from NADP^+^ as well as nucleotide precursors. The expression of *G6pdx*, which catalyzes the rate-limiting step of the oxidative PPP, was elevated after CG-5 treatment. Other genes involved in PPP such as *Rpe* was also increased in CG-5 treated-cells (Figure 2C). *Pgd*, another gene involved in PPP, showed a slight increase in CG-5 treated cells that did not reach significance. In agreement with the increased expression of PPP genes, CG-5 treated cells exhibited a decreased NADP^+^/NADPH ratio, which indicated an enhanced NADPH production by an activated PPP (Figure 2D). Genes involved in glutaminolysis such as *Odc* and *Gls2* were not affected by CG-5 (Supplemental Figure 3), suggesting that glutamine oxidation did not compensate for the reduction of glucose flux. Taken together, our results indicate that inhibition of glucose transport enhances FAO and PPP in activated CD4^+^ T cells, the former as an alternative energy source and the latter to prioritize the reduced glucose flux for anabolic pathways.

### CG-5 Inhibits Th1 and Th17 Differentiation While Promoting Treg Differentiation

**Figure 3:**
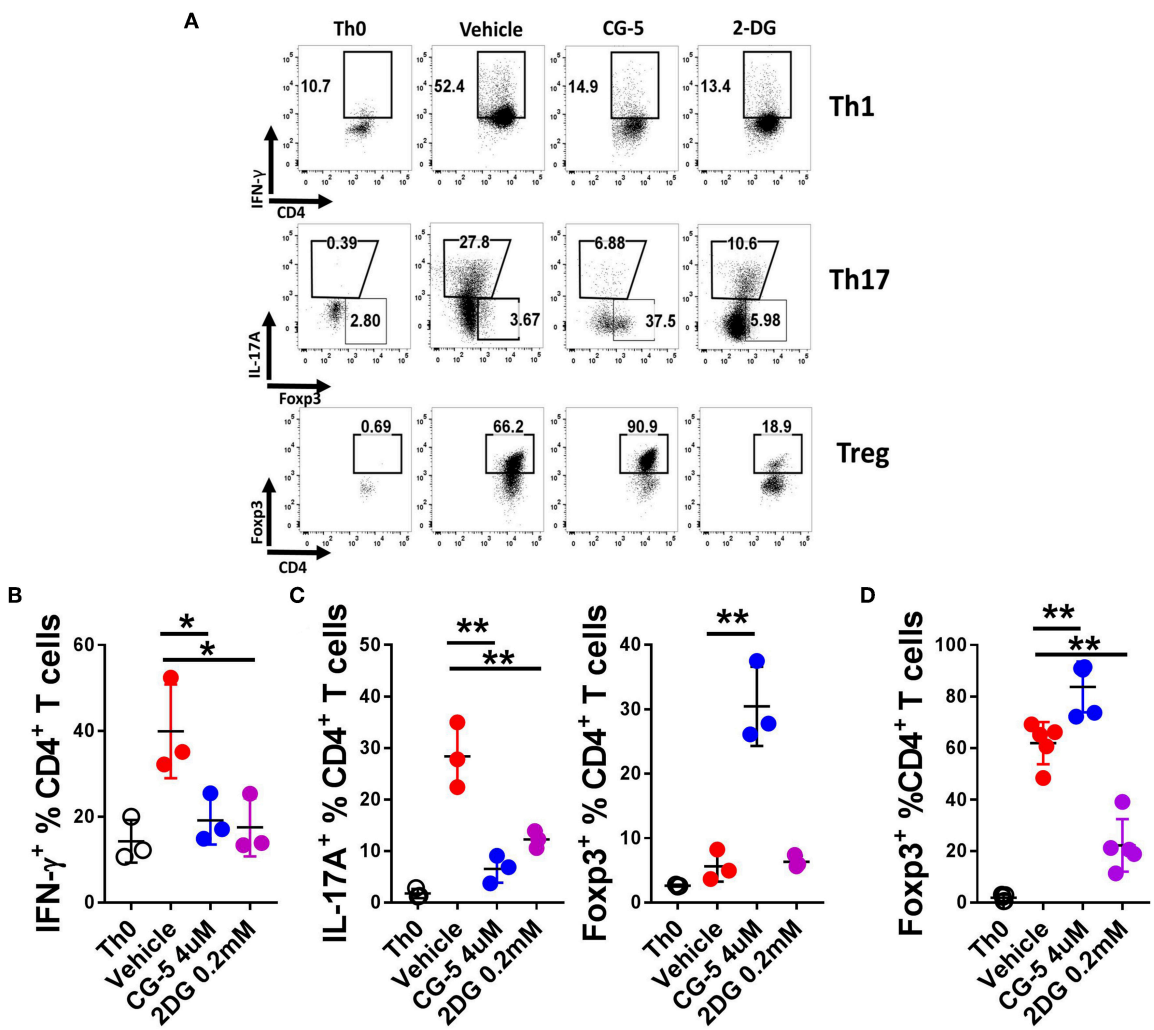
CG-5 inhibits Th1 and Th17 while promoting Treg differentiation *in vitro*. CD4^+^ T cells from B6 mice were cultured under polarizing conditions in the presence of vehicle, CG-5, or 2DG for 5 days. **(A)** Representative FACS plots of live CD4^+^-gated cells stained with IFN-γ (Th1), IL-17A and Foxp3 (Th17), and Foxp3 (Treg). Th0 conditions only contain antiCD3/CD28 mAbs. Frequency of IFN-γ ^+^CD4^+^ T cells under Th1 conditions **(B)**, IL-17A^+^CD4^+^ and Foxp3^+^CD4^+^ under Th17 conditions **(C)**, and Foxp3^+^CD4^+^ under Treg conditions **(D)**. Mean ± S.E.M of *n* = 3–5 per group compared with the vehicle-treated group using one-way ANOVA. *p < 0.05; **p < 0.01.

T cell activation initiates a transition from quiescence to rapid cell growth, proliferation, and differentiation into functional subsets to either drive or suppress the immune response (24). To address the role of CG-5 in T cell differentiation, we activated naive T cells under Th1, Th17, and Treg cells polarizing conditions *in vitro* and assessed the expression of IFN-γ, IL-17A, and Foxp3, respectively, to assess their differentiation. Treatment with CG-5 reduced the percentage of Th1 and Th17 cells in a similar manner as 2DG (Figures 3A–C). Interestingly, an increased percentage of Foxp3^+^ cells were detected in the CG-5-treated group in the Th17-polarizing conditions, suggesting a shift of Th17 to Treg cells when glycolysis is blocked (Figure 3C). Finally, CG-5 increased Treg polarization (Figure 3D), indicating Treg differentiation is favored by non-glycolytic conditions. These results are consistent with glycolysis being required for Teff, but not Treg cell expansion (10). The lack of Treg cell expansion with 2DG may indicate that in Treg inhibition of glycolysis by this drug may be counterbalanced by its inhibition of N-glycosylation, which is required by Treg cells (25).

### CG-5 Inhibits CD4^+^ T Cell Expansion in Mixed Lymphocyte Reaction

**Figure 4:**
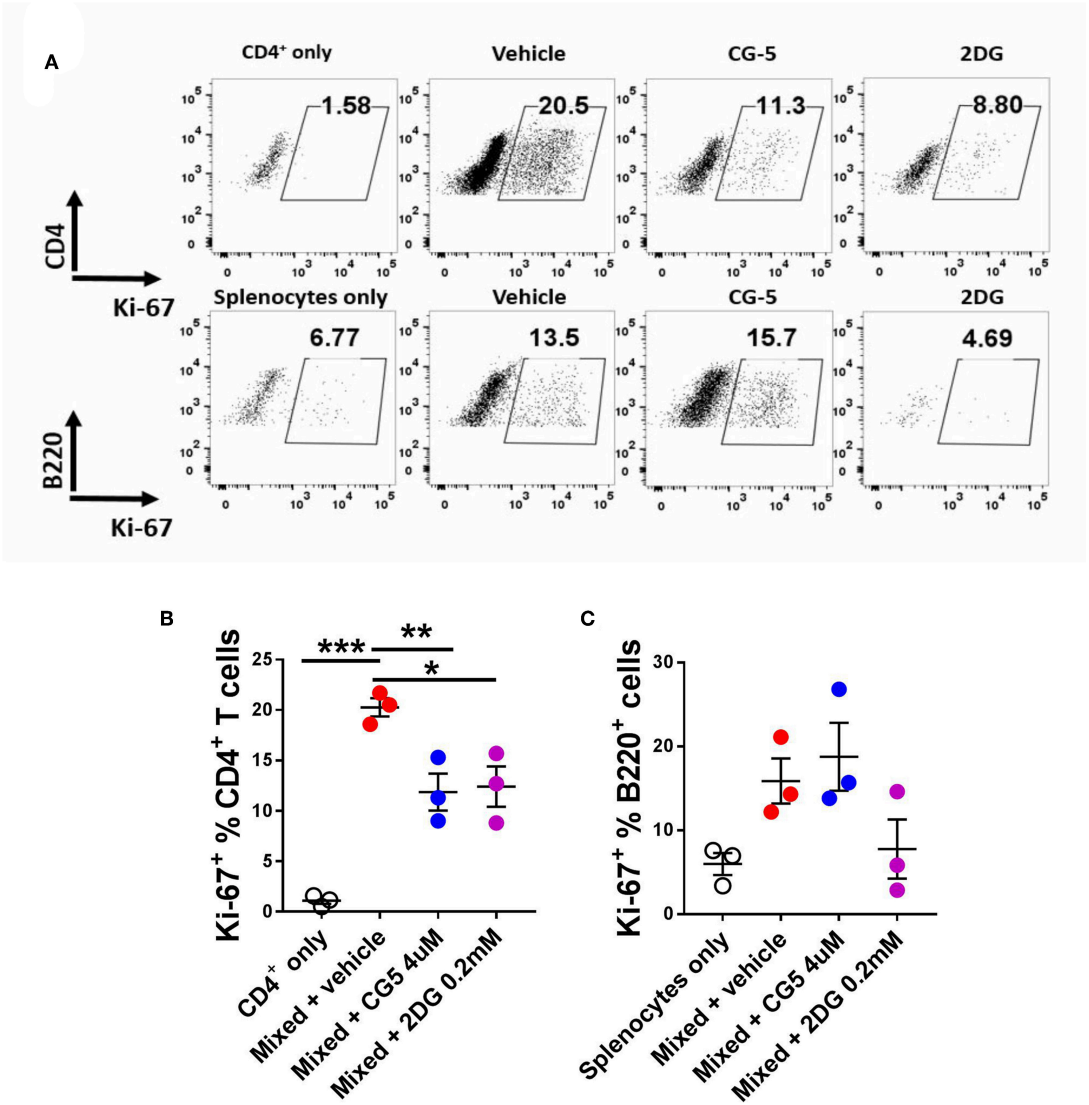
CG-5 inhibits CD4^+^ T cell alloreactivity. CD4^+^ T cells isolated from Bm12 mice were mixed with splenocytes from TCRβ KO mice in the presence of vehicle, CG-5, or 2DG for 4 days. **(A)** Representative FACS plots. Frequency of Ki-67 expression in CD4^+^ T cells **(B)** and B220^+^ B cells **(C)**. Mean ± S.E.M of *n* = 3–5 per group compared with mixed + vehicle-treated group using one-way ANOVA. *p < 0.05; **p < 0.01; ***p < 0.001.

To further evaluate the effect of CG-5 on T cell activation, we used the mixed lymphocyte reaction (MLR) model. We mixed CD4^+^ T cells from Bm12 mice with splenocytes from TCRβ KO mice, which induce the proliferation of CD4^+^ T cells through a three amino acid mismatched MHC class II molecule (26). TCRβ KO mice are deficient in T cells, therefore all the CD4^+^ T cells were from Bm12 origin. After 4 days, the vehicle-treated group showed an increased frequency of proliferating Ki-67^+^CD4^+^ T cells as compared with the non-mixed group, indicating alloreactivity-induced proliferation. The CG-5 and 2DG treatments reduced the frequency of proliferating CD4^+^ T cells (Figures 4A,B). However, CG-5 did not affect the frequency of proliferating B cells (Figure 4C). 2DG, but not CG-5, showed a trend toward reduced B cell proliferation. The results may indicate that in these *in vitro* MLR conditions, B cells, which are modestly activated, are not affected by the inhibition of glucose uptake but relatively more by the inhibition of glucose utilization. Comparatively, T cells are strongly activated and respond to both. Additional experiments will be necessary to test this hypothesis. Overall, our results show that CG-5 inhibits alloreactivity-induced activation and proliferation in CD4^+^ T cells.

### CG-5 Treatment Ameliorates Lupus Phenotypes in Mouse Models

**Figure 5:**
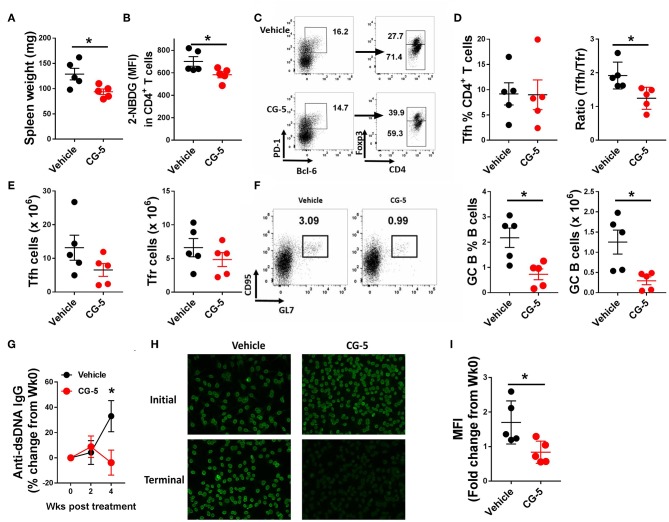
CG-5 ameliorates lupus-phenotypes in TC mice. TC mice were treated with CG-5 or vehicle at 100 mg/kg/day for 1 month. **(A)** Spleen weight. **(B)** Glucose uptake in CD4^+^ T cells measured as 2-NBDG MFI. **(C)** Representative FACS plots of Tfh cells (PD-1^hi^BCL-6^+^Foxp3^−^) and Tfr cells (PD-1^hi^BCL-6^+^Foxp3^+^). **(D)** Frequency of Tfh cells, and ratio of Tfh/Tfr. **(E)** Number of Tfh and Tfr cells. **(F)** Representative FACS plots, frequency and number of GC B (B220^+^CD95^+^GL7^+^) cells. **(G)** Time course analysis of serum anti-dsDNA IgG levels after CG-5 treatment, normalized to week 0 for each individual mouse. **(H)** Represent of ANA staining with initial (week 0) and terminal (week 4) serum from vehicle or CG-5 treated mice, and fold changes of fluorescence intensity **(I)**. Mean ± S.E.M from *n* = 5 mice per group compared with *t*-test. *p < 0.05.

Given that inhibition of glycolysis through CG-5 suppresses CD4^+^ T cell polarization and alloreactive activation *in vitro*, we next examined whether inhibition of glucose transport affects the progression of autoimmune phenotypes in lupus-prone mice. We treated 6–7 months-old TC mice with CG-5 or vehicle by gavage for 1 month. This age is an early stage of clinical disease, in which TC mice show splenomegaly, anti-dsDNA IgG production, and accumulation of activated Tfh cells and B cells (3). CG-5 treatment significantly reduced splenomegaly (Figure 5A) as well as the glucose uptake in CD4^+^ T cells (Figure 5B). The number of total CD4^+^ T cells decreased in CG-5-treated TC mice, however the frequency of Tn effector memory T cells (Tem) and Treg cells were not affected (data not shown). The frequency of Tfh cells was also unchanged while their total number showed a slight decrease in the CG-5-treated group (Figures 5C–E). However, there was a decreased ratio of Tfh to follicular regulatory (Tfr) cells (Figure 5D), which was also observed in 2DG treated TC mice (16). The high frequency and number of GC B cells in TC mice was also significantly reduced by CG-5 (Figure 5F). Consistent with the effect on GC B cells, serum anti-dsDNA IgG (Figure 5G) and anti-nuclear autoantibodies (ANA) (Figures 5H,I) were also reduced after 1 month-treatment with CG-5. Taken together, our results suggest that CG-5 ameliorates autoimmune phenotypes in TC mice.

**Figure 6:**
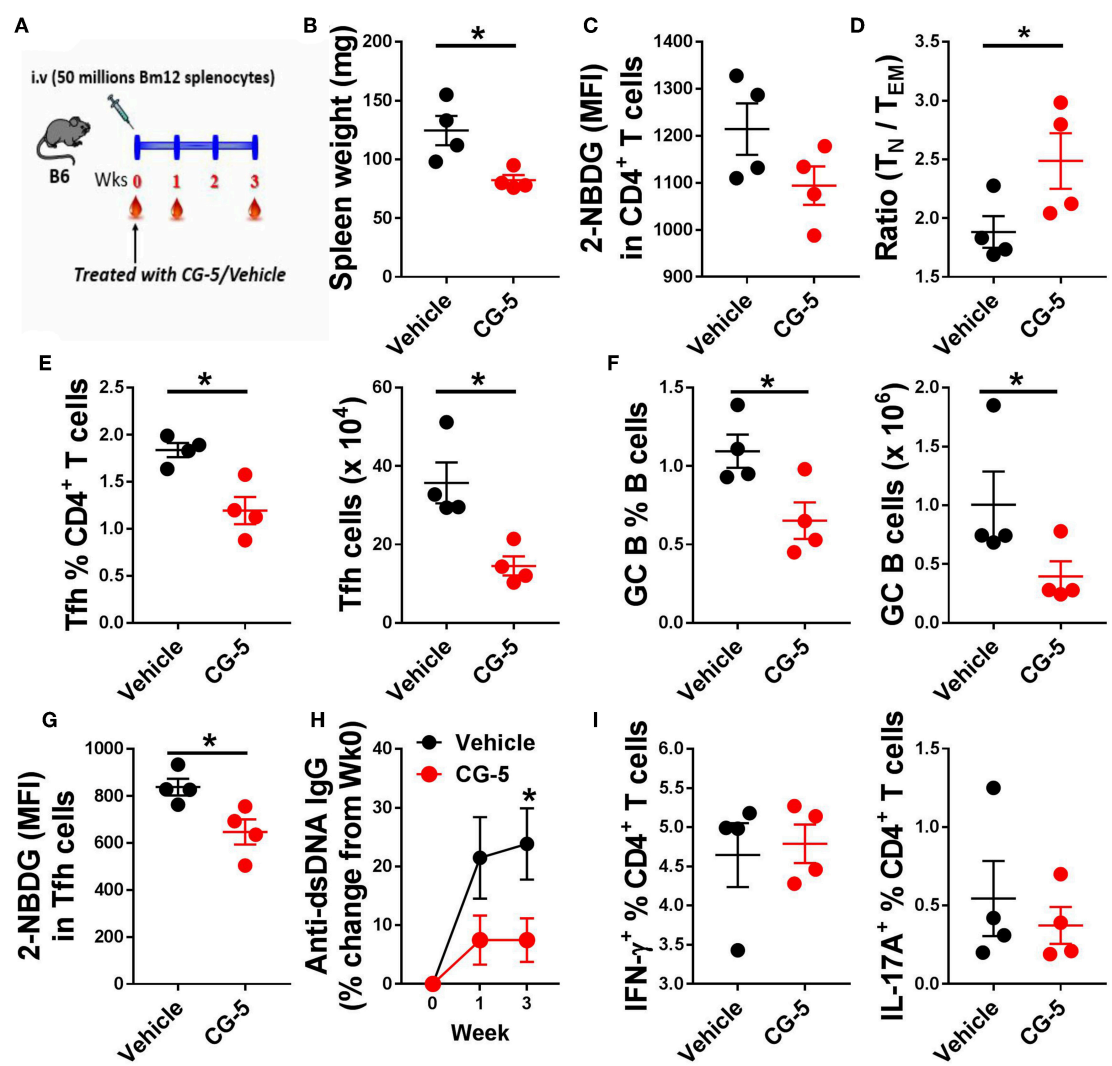
CG-5 reduces cGVHD induced autoimmunity. Phenotypes were analyzed 3 weeks after cGVHD induction with bm12 splenocytes in B6 mice treated with vehicle or CG-5. **(A)** Experimental design. **(B)** Spleen weight. **(C)** Glucose uptake in CD4^+^ T cells measured as 2-NBDG MFI. **(D)** Ratio of naïve (T_N_)/effector memory (T_EM_) CD4^+^ T cells. Frequency and number of Tfh cells **(E)** and GC B cells **(F)**. **(G)** 2-NBDG MFI in Tfh cells. **(H)** Serum anti-dsDNA IgG normalized to week 0. **(I)** Frequency of IFN-γ or IL-17A-positive CD4^+^ T cells in splenocytes from treated mice and controls. Mean ± S.E.M of *n* = 4 per group compared with *t*-tests. *p < 0.05.

We next investigated whether CG-5 treatment could alleviate cGVHD responses, a model of systemic autoimmunity by injecting Bm12 splenocytes into the B6 recipient mice (Figure 6A). In this model, donor CD4^+^ T cells react to mismatched MHC II on host B cells triggering the polyclonal activation of autoreactive B cells and, eventually, a lupus-like syndrome (26). At week 3, we found a markedly decreased spleen weight in mice received CG-5 compared to controls (Figure 6B). Similar to the TC model, CD4^+^ T cells from CG-5-treated mice showed a decreased glucose uptake (Figure 6C). CG-5-treated mice exhibited decreased number of total spleen cells as well as CD4^+^ T cells (not shown), indicating that CG-5 inhibits CD4^+^ T cell expansion in GVHD responses, in agreement with the previous alloreactivity experiment (Figure 4). Further, CG-5 treated mice showed an increased ratio of Tn/Tem cells (Figure 6D), as well as a reduced frequency of Tfh and GC B cells (Figures 6E,F). Importantly, the glucose uptake in these Tfh cells was also decreased by CG-5 (Figure 6G). Finally, CG-5 treatment also lowered serum anti-dsDNA antibody production (Figure 6H). However, we did not find any differences in the frequency of Th1 or Th17 cells (Figure 6I). Overall, our results indicate autoimmune activation was reduced in the cGVHD mice treated with CG-5.

### CG-5 Inhibits Glycolysis in Human CD4^+^ T Cells

**Figure 7:**
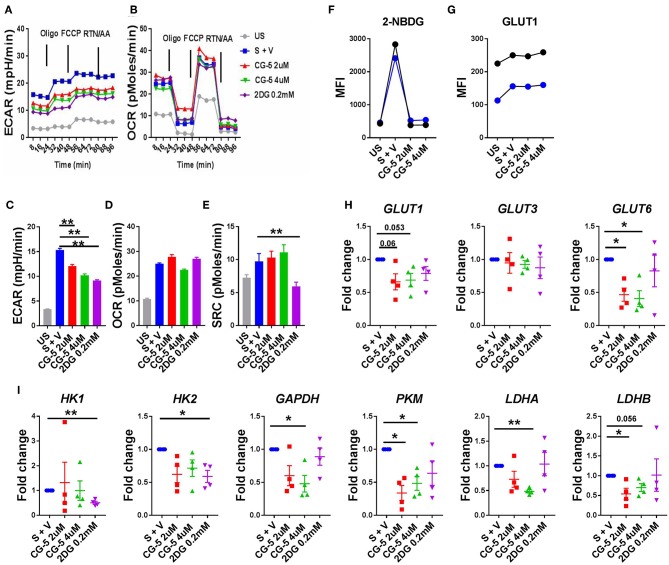
CG-5 blocks glycolysis in human CD4^+^ T cells. ECAR **(A)** and OCR **(B)** were measured in human CD4^+^ T cells, either unstimulated (US) or stimulated with anti-CD3/CD28 mAbs (S) for 24 h in the presence of vehicle (V), 2 or 4 μM CG-5, or 0.2 mM 2DG. Quantification of basal ECAR **(C)**, OCR **(D)** and SRC **(E)**. 2-NBDG uptake **(F)** and GLUT1 protein expression **(G)** in stimulated human CD4^+^ T cells (*n* = 2) for 24 h in the presence of 2 or 4 μM CG-5. **(H,I)** Expression of glycolytic genes normalized to vehicle-treated group. Mean ± S.E.M. of *n* = 4 per group compared with S + V values using one-way ANOVA. *p < 0.05; **p < 0.01.

After confirming that the CG-5 blocked glycolysis in murine CD4^+^ T cells both *in vivo* and *in vitro*, we evaluated the effect of CG-5 in human CD4^+^ T cells. CD4^+^ T cells from healthy donors were stimulated with anti-CD3/anti-CD28 Abs in the presence of CG-5 or 2DG. After 24 h, the cell viability remained 90% in all groups (not shown). In agreement with the data obtained with murine CD4^+^ T cells, CG-5 treatment significantly decreased the ECAR but not the OCR and SRC in human CD4^+^ T cells (Figures 7A–E). Also similar to murine CD4^+^ T cells, CG-5 blocked glucose uptake in activated human CD4^+^ T cells (Figure 7F). However, CG-5 did not impact GLUT1 protein expression, at least during a 24 h interval (Figure 7G), which was sufficient to decrease mouse Glut1 expression (Figure 1K). We next investigated whether CG-5 affected the expression of glycolytic genes comparatively to 2DG. CG-5 treatment lowered the expression of its target *GLUT1*, as well as that of *GLUT6* but not *GLUT3*, while no significant differences were obtained with 2DG (Figure 7H). Conversely, 2DG decreased the expression of its targets *HK1* and *HK2*, encoding for the hexokinases that control the first step of glycolysis, while CG-5 had no effect on these genes (Figure 7I). CG-5 however decreased the expression of enzymes involved in the latter steps of glycolysis, such as *GAPDH* and *PKM* with a strong inhibition of the genes encoding for lactate dehydrogenases (*LDHA* and *LADHB*) that are necessary for non-aerobic glycolysis (Figure 7I). Taken together, these results demonstrate that CG-5 inhibits glucose uptake and glycolysis in human CD4^+^ T cells, with a more complex effect on the expression of glycolytic genes, probably as a consequence of the decreased glucose flux.

## Discussion

The facilitative Glut proteins regulate the availability of glucose in most tissues. Among the 13 Glut family members, Glut1, the most widely expressed facilitative transporter, regulates basal glucose uptake in most tissues and plays an important role in cell proliferation and functions (27, 28). Given the dependence of many cancers on Glut1 transport activity, there is strong interest in Glut1 inhibitors as potential therapeutics. These inhibitors include natural compounds such as quercetin (29, 30). Quercitrin, a derivative compound of quercetin, attenuated lupus phenotypes in a GVHD mouse model (13), and one of the many targets of quercitrin may be glucose uptake. High affinity Glut 1 inhibitors such as WZB-117 (31–33) and BAY-876 (34) have been newly synthesized. Relevant to autoimmunity, treatment with WZB-117 reduced the induction of psoriasis by imiquimod or IL-23 injections by limiting glucose uptake in keratinocytes (33). However, to our knowledge, the effect of these inhibitors has yet to be tested in immune cells. Here, we showed with several *in vitro* measurements, including glycolytic gene expression, metabolic assays, and glucose uptake demonstrated that glycolysis was blocked by CG-5 in both mouse and human CD4^+^ T cells. We obtained mixed results regarding the effect of CG-5 on the expression of the glucose transporters themselves: CG-5 reduced *Glut1* and *Glut6* mRNA expression in human but not in mouse T cells, but Glut1 protein levels were decreased in CG-5 treated mouse but not human T cells. The effect of glucose transporter or glycolysis inhibitors on the expression of glycolytic genes has not been reported to the best of our knowledge. However, it represents a secondary effect of the inhibition of glucose flux, and its significance is unclear, since in spite of discrepancies between species regarding gene expression, glycolysis was functionally inhibited. Further studies will be required to address this issue.

In the past few years, cellular metabolism has been under the spotlight for its pivotal role in inflammatory and autoimmune diseases. Immune cells adapt their metabolic status as a consequence to changes in the external microenvironment. T cells are the key players of adaptive immunity and show high metabolic demand for the activation, proliferation, differentiation, and migration to target organs. In autoimmune diseases, autoreactive CD4^+^ T cells show elevated metabolism with increased glycolysis and mitochondrial respiration (3, 15). 2DG, a commonly used glycolysis inhibitor, has been used in various mouse models. For instance, inhibiting glycolysis with 2DG and glutaminolysis with DON (6-diazo-5-oxo-L-norleucine) prevented allograft rejection (35), and 2DG attenuated autoimmune phenotypes in lupus-prone mouse models (16). 2DG also reduced joint inflammation and the activation of adaptive and innate immune cells in a rheumatoid arthritis model (15). Other glycolytic inhibitors, such as 3-bromopyruvate (BrPA), a specific HK2 inhibitor decreased arthritis score and histological scores in the SKG mouse model (36). 3PO, a small molecule inhibitor of PFKFB3, an enzyme that controls a rate-limiting step of glycolysis, prevented the development of T-cell mediated delayed hypersensitivity and imiquimod-induced psoriasis in mouse (37). Dimethyl fumarate, a derivative of the Krebs cycle intermediate fumarate that inactivates glyceraldehyde 3-phosphate dehydrogenase (GAPDH), attenuated Th1 and Th17 responses in the experimental autoimmune encephalomyelitis model (38). These results, combined with our results obtained with CG-5, represent a body of evidence supporting glucose inhibition as a therapeutic approach for the treatment of autoimmune diseases, including SLE.

Teff or activated T cells increase glycolysis and mitochondrial metabolism to meet biosynthetic demands while FAO is essential for the development and survival of memory T cells and Treg cells *in vitro* (11). Consistently with these findings, CG-5 promoted FAO and increased Treg differentiation under polarizing conditions *in vitro*. Interestingly, the frequency of Treg cells were decreased in the CG-5 treatment group in the cGVHD model (data not shown). Treg frequency was not affected in 2DG-treated TC lupus-prone mice in spite of a reduction of disease severity (3, 15). The *in vivo* metabolic requirements of Treg cells in the context of autoimmune activation have not yet been defined. Globally, our results suggest that the frequency of Treg cells is not affected by glucose inhibition *in vivo* as it is *in vitro*, although we cannot exclude at this point that it enhances their suppressive function. The expansion of germinal centers with GC B cells and Tfh cells is necessary to produce high levels of anti-dsDNA IgG. A new Treg cell subtype named Tfr has recently been described and provides a better understanding of the role of Treg cells in GC reactions. Tfr cells negatively regulate the expansion or function of the Tfh population (39, 40). Further, excessive Tfh cell number are found in lupus-prone mice and increased circulating Tfh cells are found in lupus patients (41), indicating an essential role of Tfh cells in SLE. In addition, the Tfh/Tfr ratio correlated with disease activity in SLE patients (42). In the cGVHD mouse model, frequency and number of Tfh cells were reduced as well as of the GC B cells, along with the production of anti-dsDNA IgG. Unlike cGVHD model, the percentage of Tfh did not change in TC mice, but the ratio of Tfh/Tfr decreased in the CG-5 group. There is robust evidence that autoimmune T cells in lupus disease have an abnormal metabolism. Recently, our group demonstrated that in the TC mouse model of lupus, spontaneous Tfh cells have increased glucose requirements compared to pathogen-induced Tfh cells (16). CG-5 decreased the glucose uptake by Tfh cells, which might lead to the decreased Tfh cell number in the GVHD model. Overall, this study showed that the inhibition of glucose transporters with CG-5 significantly attenuates autoimmune activation in the TC and cGVHD models of SLE, at least in part through an effect on CD4^+^ T cells. The purpose of the current study was to test whether blocking glucose uptake with CG-5 would have the same effect as blocking HK with 2DG in mouse models of lupus. Since most of the data we have published with 2DG, focused on CD4^+^ T cells (14, 16), CD4^+^ T cells were also the main focus of this paper and we showed that CG-5 affects CD4^+^ T cell functions. Its effect on B cells was less salient. CG-5 failed to inhibit B cell proliferation in *in vitro* allo reaction experiments. *In vivo*, we did not find a decreased glucose uptake in non-CD4^+^ T cells (which are in large majority B cells). Though CG-5 showed a strong effect on GC B cells, we cannot ascertain whether it was a direct effect on GC B cells, or an indirect effect through Tfh cells or other cell types. Therefore, while focused on CD4^+^ T cells, our results do not exclude that CG-5 has additional effects on other immune cell subsets, including B cells and dendritic cells. Regardless of the cellular target, our results suggest targeting glucose transporters present a potential therapeutic strategy in immune metabolic reprogramming for the treatment of SLE.

## Ethics Statement

UF IRB approval IRB201700257.

## Author Contributions

WL designed, performed experiments, and wrote the manuscript. GQ, CC, AT, and NK performed experiments. S-CC, XT, and HW contributed reagents and participated to experimental design and data interpretation. LM conceived the study, participated to experimental design and interpretation, and co-wrote the manuscript.

### Conflict of Interest Statement

The authors declare that the research was conducted in the absence of any commercial or financial relationships that could be construed as a potential conflict of interest.

## Abbreviations

2DG: 2-deoxy-D-glucose
cGVHD: chronic graft- vs. host-disease
Treg: regulatory T cells
GC: germinal center
Tfh: follicular helper T cells
Tfr: T follicular regulatory cells
Tn: Naïve T cells
Teff: effector T cells
OXPHOS: oxidative phosphorylation
OCR: oxygen consumption rate
ECAR: extra cellular acidification rate
FAO: fatty acid oxidation
PPP: pentose phosphate pathway
ANA: anti-nuclear autoantibodies
SLE: systemic lupus erythematosus.
