# Supplementary material for: Targeting T Cell Activation and Lupus Autoimmune Phenotypes by Inhibiting Glucose Transporters
Source: Front Immunol. 2019 Apr 17;10:833. doi: 10.3389/fimmu.2019.00833 (PMC6478810; doi:10.3389/fimmu.2019.00833)
Supplement: Supplementary file 1 [file Data_Sheet_1.PDF]

Supplemental Table 1. Primers used for qRT-PCR.

Mouse	
Glut1	Forward: 5'-CAGTTCGGCTATAACACTGGTG-3'
	Reverse: 5'-GCCCCCGACAGAGAAGAT G-3'
Glut3	Forward: 5'-GCTTTGGCAGACGCAACTCT-3'
	Reverse: 5'-TAGTGTGCCAAATGCACCCC-3'
Glut6	Forward: 5'-CTCTACGCCCTTGGTCTGC-3'
	Reverse: 5'-ATCATGATGAGAACCGGCCC-3'
Ldha	Forward: 5'-CATTGTCAAGTACAGTCCACA CT-3'
	Reverse: 5'-TTCCAATTACTCGGTTTTTGGGA-3'
Ldhb	Forward: 5'-GATTCCGCTACCTCATGGCA-3'
	Reverse: 5'-ATGCCGTACATTCCCTGTCC-3'
Hk1	Forward: 5'-TTCGAGAAGATGGTGAGCGG-3'
	Reverse: 5'-GGAGAGTTCCCATCCCGTTT-3'
Hk2	Forward: 5'-TGATCGCTGCTTATTCACGG-3'
	Reverse: 5'-AACCGCCTAGAA ATCTCCAGA-3'
Cpt1-b	Forward: 5'-CGTTCCTGTACCAACGAGTC-3'
	Reverse: 5'-CAGAAAGTACCTCAGCCAGG-3'
Pkm	Forward: 5'-GCCGCCTGGACATTGACTC-3'
	Reverse: 5'-CCATGAGAGAAATTCAGGCGAG-3'
Gls2	Forward: 5'-AGCGTATCCCTATCCACAAGTTCA-3'
	Reverse: 5'-GACGTCCAGTGGCCTTCAGAG-3'
Cpt1a	Forward: 5'-CCAGGCTACAGTGGGACATT-3'
	Reverse: 5'-GAACTTGCCCATGTCCTTGT-3'
G6pdx	Forward: 5'-CACAGTGGACGACATCCG AAA-3'
	Reverse: 5'-AGCTACATAGGA ATTACGGGC AA-3'
Rpe	Forward: 5'-GAAGTGTGCGGAGGCAG-3'
	Reverse: 5'-TTTAATCACTGGAATGCAGCAC-3'
Cpt2	Forward: 5'-ACCATGCACTACCAGGACAG-3'
	Reverse: 5'-TATCAAACCAGGGGCCTGAGA-3'
Odc	Forward: 5'-GACGAGTTTGACTGCCACATC-3'
	Reverse: 5'-CGCAACATAGAACGCATCCTT-3'
Pgd	Forward: 5'-CGTAAGGCCCTCTATGCTTC-3'
	Reverse: 5'-TGAAGTTCTGGGTTTCGCTC-3'
Gapdh	Forward: 5'-GAAACTGGGAAGGGGAGTGG-3'
	Reverse: 5'-GCCAAATCCGTTCACACCG-3'
Ppia	Forward: 5'-GCTGTTTGCAGACAAAGTTCCA-3'
	Reverse: 5'-CGTGTAAAGTCACCACCCTGG-3'

Human	
GLUT1	Forward: 5’-TTGGCTCCGGTATCGTCAAC-3’
	Reverse: 5’-CTCCACCACAAACAGCGACA-3’
GLUT3	Forward: 5’-GTCATGATCCCAGCGAGACC-3’
	Reverse: 5’-CTGGGGTGACCTTCTGTGTC-3’
GLUT6	Forward: 5’-CCATGCTCTTCATCATGGGCTAC-3’
	Reverse: 5’-CACTGGCAGGAAGGACTTGG-3’
LDHA	Forward: 5’-TGGAGATTCCAGTGTGCCTG-3’
	Reverse: 5’-CCTGCTTGTGAACCTCTTTCC-3’
LDHB	Forward: 5’-GGAGAGTCGGCTCAATCTGG-3’
	Reverse: 5’-AGAATGTCCACTGGGTTGGA-3’
HK1	Forward: 5’-TAAAGCGAGCGGAGTGGAAG-3’
	Reverse: 5’-ACAGTGCTGGTCGTCATAGC-3’
HK2	Forward: 5’-ATTGCCGAATGCCTGGCTAA-3’
	Reverse: 5’-CAAAGTCCCCTCTCCTCTGGA-3’
PKM	Forward: 5’-ATCTGCACAACCTGTGACCG-3’
	Reverse: 5’-ATAAACACCCGACGCTTGGT-3’
GAPDH	Forward: 5’-GCAAATTCCATGGCACCGTC-3’
	Reverse: 5’-AAATGAGCCCCAGCCTTCTC-3’
PPIA	Forward: 5’-AGCTGTTTACCCCTGATCGTG-3’
	Reverse: 5’-CCTTGTCTGCAAACAGAAGGC-3’

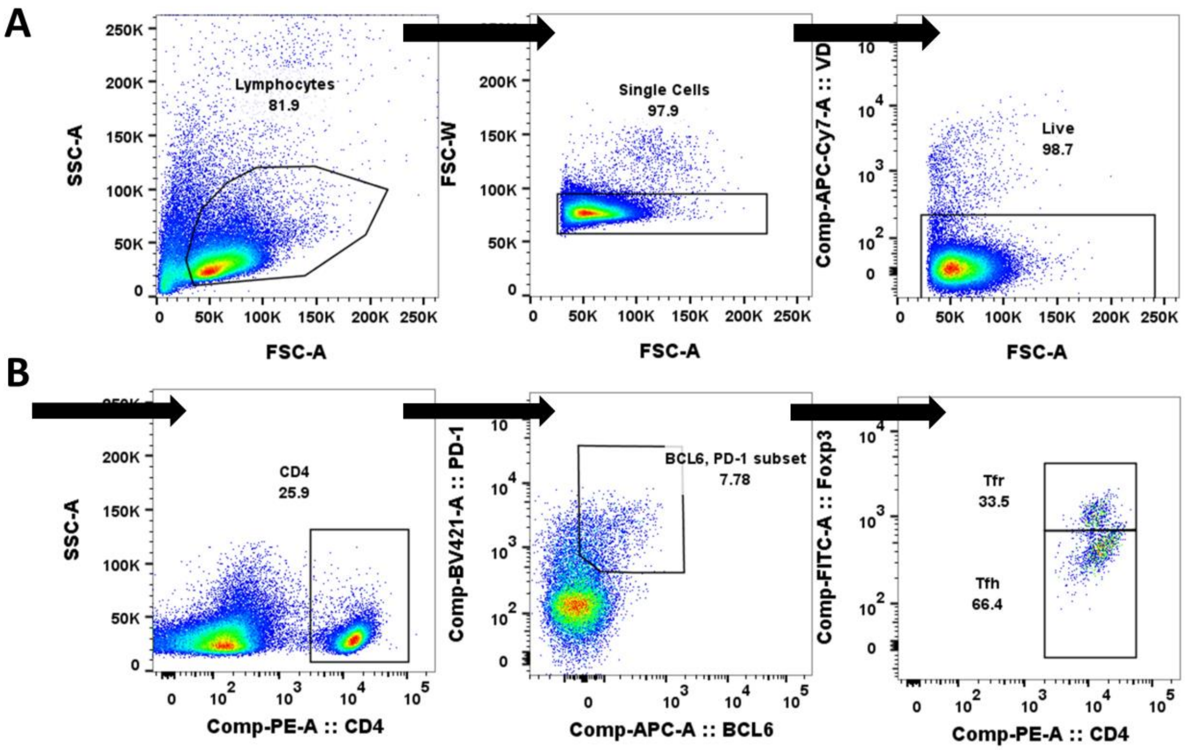

Sup Fig. 1. Gating strategy. (A) Live lymphocytes gating. Lymphocytes were gated based on side-scatter area (SSC-A) and forward scatter area (FSC-A), followed with single cells gating by FSC-W and FSC-A. Then live cells were identified by viability dye (VD) exclusion. **(B)** Gating for Tfh (follicular T helper) and Tfr (follicular regulatory T) cells. Among the live cells gated in **(A)**, the $CD4^+$ T cells were gated, following with $BCL6^+$ $PD\text{-}1^+$ follicular cells, among which the $Foxp3^-$ cells are identified as Tfh and $Foxp3^+$ as Tfr cells.

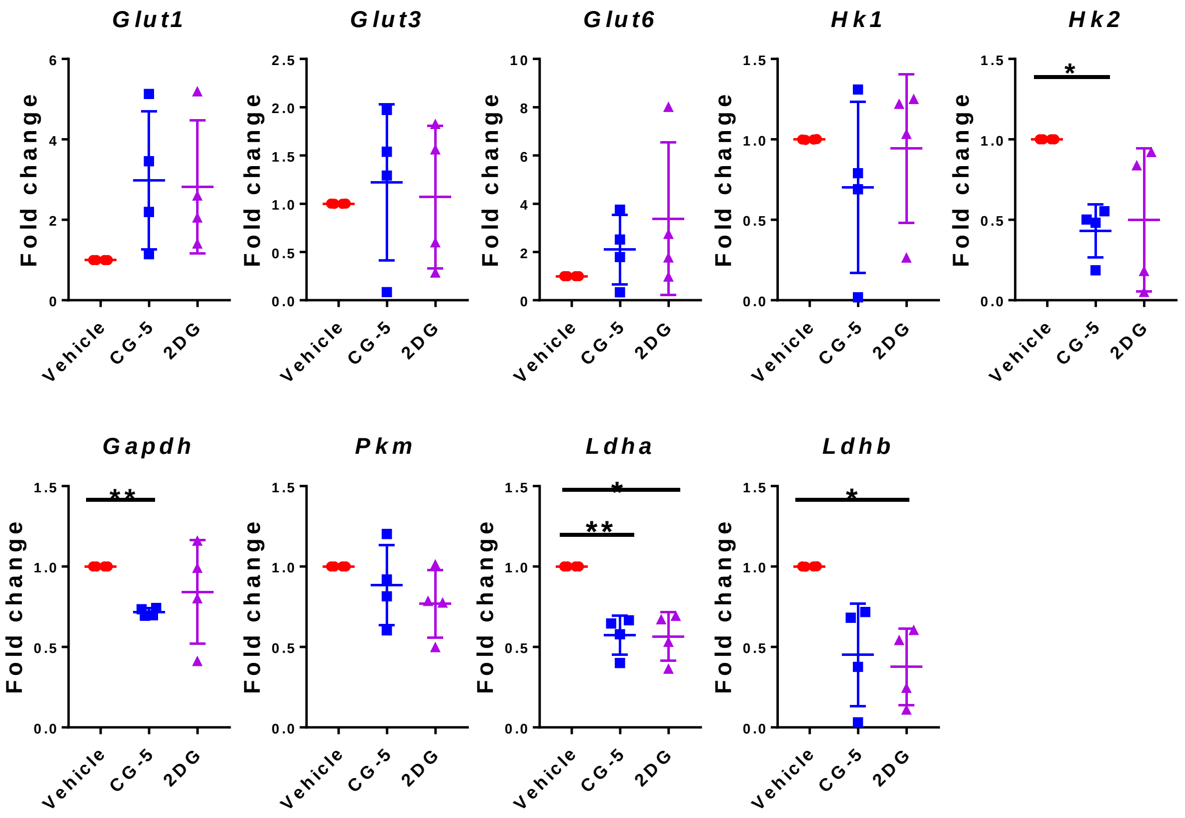

Sup. Fig. 2. Glycolytic gene expression in CG-5 treated CD4+ T cells.

CD4+ T cells from B6 mice were stimulated with anti-CD3/CD28 for 24 h with or without 4 uM CG-5 or 0.2 mM 2DG. The relative expression of glycolytic genes was normalized to the vehicle-treated values in every single mouse. Results expressed as means ± S.E.M from n = 4 per group compared with the vehicle-treated group using one-way ANOVA. *: p<0.05, **: p < 0.01.

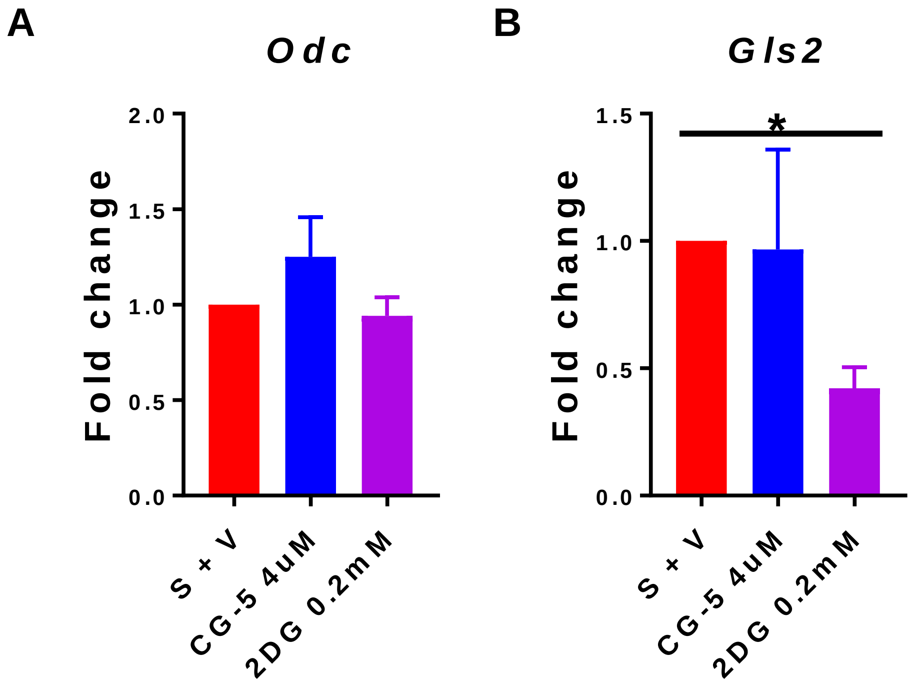

Sup Fig. 3. Glutaminolysis gene expression in CG-5 treated CD4^{+} T cells. CD4^{+} T cells from B6 mice were stimulated with anti-CD3/CD28 for 24 h with or without 4 uM CG-5 or 0.2 mM 2DG. The relative expression of *Odc* and *Gls2* was normalized to the vehicle-treated values in every single mouse. Results expressed as mean ± S.E.M from n = 4 per group compared with the S + V group using one-way ANOVA. **: $p < 0.01$.
